# Controllable permeability of blood-brain barrier and reduced brain injury through low-intensity pulsed ultrasound stimulation

**DOI:** 10.18632/oncotarget.5978

**Published:** 2015-10-16

**Authors:** Wei-Shen Su, Min-Lan Tsai, Sin-Luo Huang, Shing-Hwa Liu, Feng-Yi Yang

**Affiliations:** ^1^ Department of Biomedical Imaging and Radiological Sciences, National Yang-Ming University, Taipei, Taiwan; ^2^ Department of Pediatrics, Cheng Hsin General Hospital, Taipei, Taiwan; ^3^ Institute of Toxicology, College of Medicine, National Taiwan University, Taipei, Taiwan; ^4^ Department of Medical Research, China Medical University Hospital, China Medical University, Taichung, Taiwan; ^5^ Biophotonics and Molecular Imaging Research Center, National Yang-Ming University, Taipei, Taiwan; ^6^ Biomedical Engineering Research and Development Center, National Yang-Ming University, Taipei, Taiwan

**Keywords:** low intensity ultrasound, permeability, blood-brain barrier, edema, brain injury

## Abstract

It has been shown that the blood-brain barrier (BBB) can be locally disrupted by focused ultrasound (FUS) in the presence of microbubbles (MB) while sustaining little damage to the brain tissue. Thus, the safety issue associated with FUS-induced BBB disruption (BBBD) needs to be investigated for future clinical applications. This study demonstrated the neuroprotective effects induced by low-intensity pulsed ultrasound (LIPUS) against brain injury in the sonicated brain. Rats subjected to a BBB disruption injury received LIPUS exposure for 5 min after FUS/MB application. Measurements of BBB permeability, brain water content, and histological analysis were then carried out to evaluate the effects of LIPUS. The permeability and time window of FUS-induced BBBD can be effectively modulated with LIPUS. LIPUS also significantly reduced brain edema, neuronal death, and apoptosis in the sonicated brain. Our results show that brain injury in the FUS-induced BBBD model could be ameliorated by LIPUS and that LIPUS may be proposed as a novel treatment modality for controllable release of drugs into the brain.

## INTRODUCTION

The blood-brain barrier (BBB) is a rate-limiting factor in terms of the brain's permeability to drugs. At the same time, the BBB protects the brain from harmful substances in the blood stream. Many promising studies have demonstrated that focused ultrasound (FUS) with microbubbles (MB) can non-invasively deliver therapeutic agents to a specific region of interest in the brain through local BBB disruption (BBBD) [1–3]. Ultrasound interacts with MB to produce cavitation, which not only releases drugs, but also causes brain injury, including mild hemorrhage, edema, or apoptosis [4, 5]. Several studies have shown that FUS-induced BBBD does not cause observable histological brain damage [6, 7]. It has been shown that for specific parameters of FUS with MB can be safe for non-human primates [8, 9]. However, treatment safety depends wholly on the selection of appropriate treatment parameters Although relatively little brain damage occurs at optimum ultrasound parameters capable of FUS-induced BBBD, no investigation has showed a complete lack of brain injury when using this non-invasive technique. Therefore, investigations aimed at providing an effective method for neuroprotection against brain injury following FUS-induced BBBD are necessary.

Traumatic brain injury (TBI) occurs when a mechanical force causes brain dysfunction. Depending on the degree of injury to the brain, TBI varies from mild to moderate to severe. A recent study indicated that high-intensity FUS (HIFU) may serve as a valuable surrogate for the simulation of some bio-effect aspects of blast-related mild TBI [10]. TBI triggers a complex series of responses that contribute to neuronal death and apoptosis [11, 12]. Cerebral edema formation is the most significant predictor of outcome following TBI. It has been established that cerebral edemas can be classified into cytotoxic edemas and vasogenic edemas. Studies of rat models of TBI have showed an increase in the BBB permeability after injury [13–15]. The initial inflammatory response after TBI results in BBBD [16]. BBBD is considered to be the major cause of vasogenic brain edema and subsequent brain damage [17, 18]. There is as yet no definitive treatment method for severe brain edema after TBI, and it has become increasingly evident that cerebral edema leads to high mortality and morbidity in patients with TBI [19, 20].

An impermeable BBB makes the application of pharmacological therapy to the brain difficult, but such therapy can be vital to maintaining normal brain physiology. There may be safety concerns when the duration of BBBD lasts too long, and hence the duration of BBBD in relation to ultrasound parameters needs to be optimized. One of our previous studies demonstrated that the concentration of MB can be used to influence the duration of BBBD [21]. Moreover, another of our studies indicated the possibility of controlling how drug delivery is distributed by extending the duration of BBBD through repeated FUS sonications [22]. Other research has further suggested that ultrasound exposure in the early stages of traumatic brain injury (TBI) will not only effectively enhance recovery of the BBB, but also reduce brain edema [23].

Some studies have reported that low-intensity pulsed US (LIPUS) can be used to accelerate bone and axonal regeneration following injury [24, 25]. Furthermore, LIPUS stimulation could promote the levels of brain-derived neurotrophic factor (BDNF) in the brain [26–28]. By increasing the brain concentration of BDNF, LIPUS might play an important role in the treatment of TBI [29]. In addition, TBI in the rat model may be alleviated by ultrasound application [23]. Consequently, in the present study, we sought to investigate the hypothesis that LIPUS may modulate BBB permeability, attenuate cerebral edema, and improve histological outcomes in an experimental TBI model induced by FUS during drug delivery.

## RESULTS

### LIPUS modulated the duration of BBB disruption

**Figure 1:**
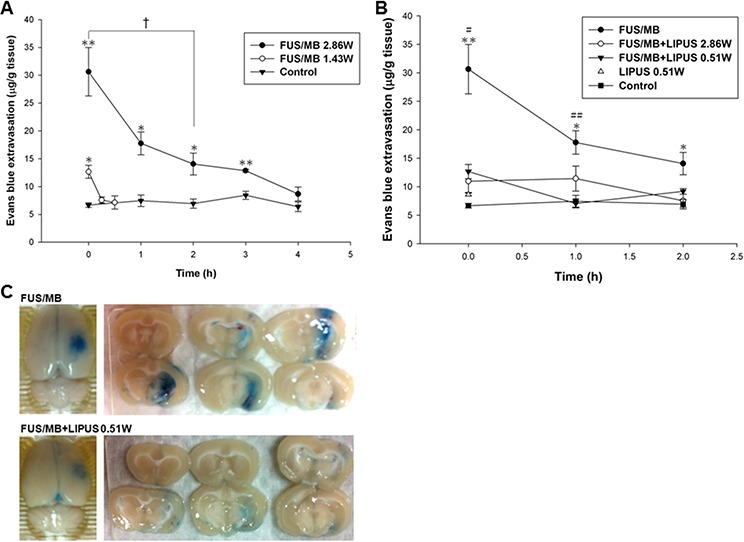
EB extravasation was assessed in the brain after sonication **A.** Graph shows the EB extravasation of 200 μL/kg UCA within 4 h after sonication in right and left (control group) brain hemispheres with and without sonication at an acoustic power of 1.43 or 2.86 W. **B.** Graph showing the amount of EB extravasation as a function of time after single or repeated sonication. EB extravasation was largest immediately after the sonications and rapidly decreased within 2 h. **C.** Distribution of BBBD for FUS/MB and FUS/MB+LIPUS group as evaluated by the extravasation of EB into the brain in the sonicated right hemisphere and the corresponding brain surface. * and # denote significantly different from control and FUS/MB+LIPUS group, respectively (*, # and †, *p* < 0.05; ** and ##, *p* < 0.01, *n* = 3).

The BBBD was quantitatively assessed in the sonicated region of the ultrasound beam with EB extravasation. Fig. 1A reveals the amount of EB extravasation in the sonicated brains at an acoustic power of 1.43 W or 2.86 W. The EB extravasation values for 1.43 W and 2.86 W showed continuous but declining BBBD and were approximately the same as the values in the contralateral brain at the time points of 30 min and 4 h after FUS/MB application, respectively. In order to investigate the effects of LIPUS treatment on the duration of BBBD, EB extravasation was therefore quantified in the sonicated brain with 2.86 W for an approximate duration of the following studies. Immediately after sonications, EB extravasation significantly decreased in the FUS/MB-sonicated brains followed by FUS (2.86 W) or LIPUS (0.51 W) application compared to the FUS/MB group (Fig. 1B). Both BBB integrities appeared to be re-established after 1 hour because injection of EB at this time led to no differences as compared with the control brain. At 1 hour after FUS/MB application, the EB staining is more broadly distributed and darker in FUS/MB group than in FUS/MB+LIPUS group (Fig. 1C). Thus, the lower power of LIPUS (0.51 W) was chosen in the following experiments to reduce the possible brain damage. Compared to the control group, there was a mild but no significant increase in the EB extravasation of the brain treated with LIPUS alone at the time point of 0 h (Fig. 1B).

**Figure 2:**
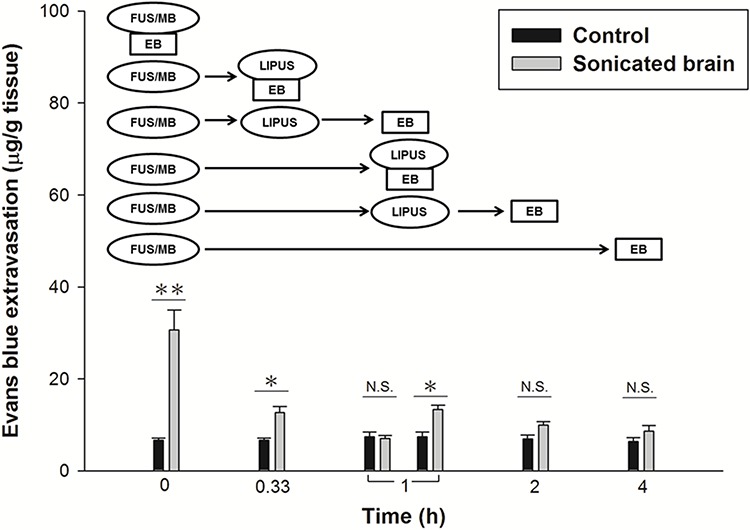
Time window evaluation for the duration of BBBD comparing a LIPUS sonication with an interval of 20 min or 1 h following the first FUS/MB application (*p < 0.05; **p < 0.01, *n* = 3). N.S. means no significance.

The evaluation of the time window for BBBD showed that significant EB extravasation occurred within the first hour, but that the extravasation had returned to baseline at 4 h after a FUS/MB application at an acoustic power of 2.86 W (Fig. 1A). A LIPUS treatment was therefore performed at 20 min and 1 h after the initial transient BBBD event, in order to explore the effect of a second LIPUS sonication on the recovery of the BBB. Immediately after the LIPUS treatment following FUS/MB application at 20 min, EB extravasation was significantly greater than for the contralateral brain (Fig. 2). Subsequently, the BBBD had recovered to baseline at 1 h after FUS/MB application. However, EB extravasation remained significantly greater than for the contralateral brain in the group with LIPUS treatment at 1 h after the first FUS/MB application. Afterward, no significant differences were found between the two hemispheres at 2 h after FUS/MB application. For both LIPUS treatment cases, the recovery time of BBB permeability was obviously decreased for the group with a single FUS/MB application.

### LIPUS attenuated brain edema

**Figure 3:**
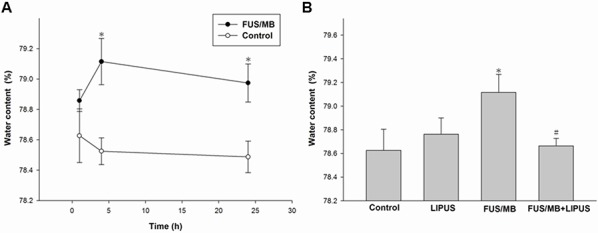
Assessment of cerebral water content induced by FUS/MB application **A.** Cerebral water contents at 1, 4, and 24 h after FUS/MB application. **B.** Water contents increased after FUS/MB and then significantly decreased after LIPUS treatment. * and # denote values significantly different from control and FUS/MB group, respectively (* and #, *p* < 0.05, *n* = 4).

Because BBBD may cause accumulation of circulating fluid and contribute to brain edema [30], we further examined whether LIPUS treatment could ameliorate brain edema. Brain water content, an indicator of brain edema, was significantly increased within 24 h and reached the maximum at 4 h in FUS/MB-treated rats compared with that of control group rats (79.1 ± 0.2% versus 78.5 ± 0.1%, *p* < 0.05; Fig. 3A). Treatment with LIPUS resulted in a reduction in the brain water content within the sonicated brains compared with the content in FUS/MB group brains at 4 h, a time point associated with maximal edema formation following FUS/MB treatment (78.7 ± 0.1% versus 79.1 ± 0.2%, *p* < 0.05; Fig. 3B). No significant difference in brain water content was found between rats treated with LIPUS alone and control group rats.

### LIPUS treatment reduced brain injury

**Figure 4:**
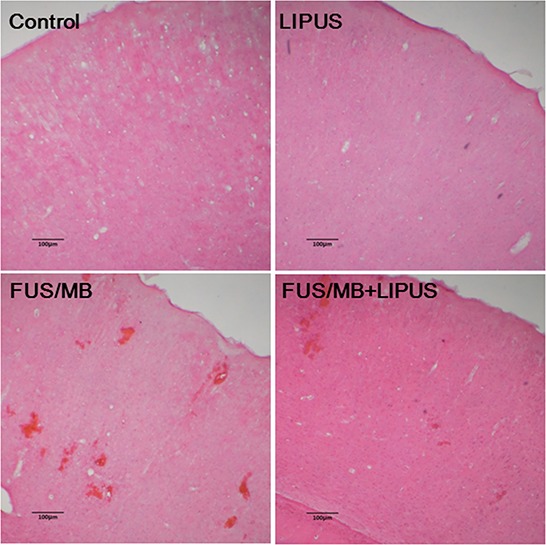
Histological evaluations with H&E staining in the brains of a control rat and of sonicated rats with LIPUS, FUS/MB, and FUS/MB+LIPUS application The scale bar is 100 μm.

Fig. 4 shows representative samples of the histologic evaluation of H&E-stained sections. No damage was observed in the brain treated with LIPUS alone. Obvious hemorrhages in the sonicated region suggest BBBD after FUS/MB application. Fewer extravasated erythrocytes were seen within the sonicated region treated with LIPUS following FUS/MB application.

**Figure 5:**
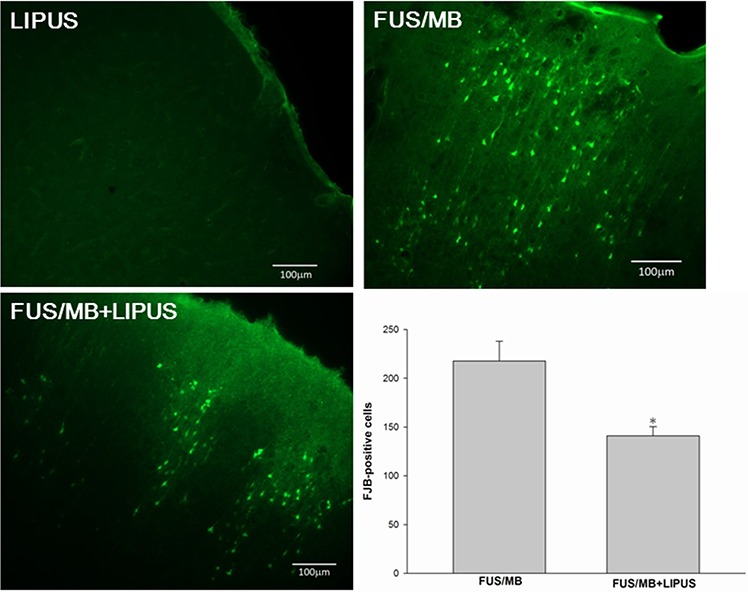
Effects of LIPUS treatment on neuronal degeneration A representative Fluoro-Jade B (FJB)-stained brain section of a sonicated region. Quantification analysis indicated that rats treated with LIPUS following FUS/MB had significantly fewer degenerating neurons than FUS/MB-treated rats. The total number of FJB-positive cells is expressed as the mean number per field of view (0.8 mm^2^). The scale bar is 100 μm. * denotes *a* value significantly different from that of the FUS/MB group (*p < 0.05, *n* = 3).

**Figure 6:**
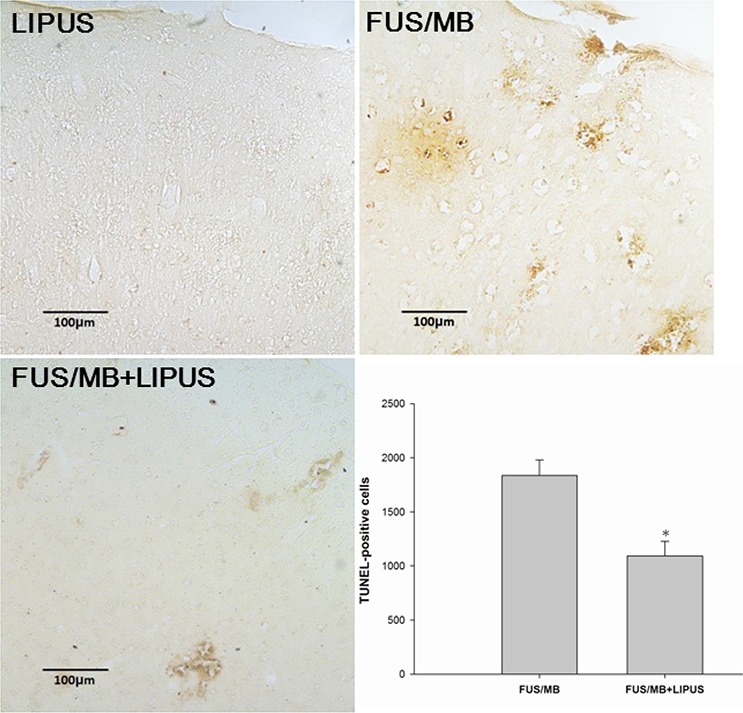
Effects of LIPUS treatment on apoptotic cell death in the sonicated brain Representative TUNEL-stained brain sections of a LIPUS-treated rat, FUS/MB-treated rat, and a LIPUS-treated rat following FUS/MB application. Quantification showed that rats treated with LIPUS following FUS/MB had significantly fewer TUNEL-positive cells than FUS/MB-treated rats. The total number of TUNEL-positive cells is expressed as the mean number in the sonicated region. The scale bar is 100 μm. * denotes *a* value significantly different from that of the FUS/MB group (*p < 0.05, *n* = 3).

FJB- and TUNEL-stained sections were used to examine whether neuronal death was decreased in the sonicated region of rats treated with LIPUS. Both FJB-positive cells with neuronal morphology and TUNEL-positive cells with apoptosis were evident at day 1 after FUS/MB application in the sonicated region but not in the group with LIPUS alone. Rats treated with LIPUS following FUS/MB had significantly fewer FJB-positive neurons in the sonicated regions at day 1 post-FUS/MB application than were observed in the FUS/MB group (140.9 ± 9.6 versus 217.7 ± 20.2 cells, *p* < 0.05; Fig. 5). Furthermore, significantly fewer apoptotic cells were found in the sonicated region treated with LIPUS compared with the FUS/MB group (1091.7 ± 135.4 versus 1836.3 ± 144.0 cells, *p* < 0.05; Fig. 6).

## DISCUSSION

This study shows for the first time that LIPUS stimulation after FUS/MB-induced BBBD modulates the duration of BBB and reduces cerebral edema. Moreover, our results demonstrate that LIPUS treatment is effective at attenuating the severity of brain injuries in rats, in terms of both neuronal damage and apoptotic cell death.

FUS-induced targeted BBBD may offer a solution to the problems associated with the delivery of drugs to the brain. Nevertheless, the duration of FUS-induced BBBD should be selective during drug delivery because the BBB protects the brain from foreign substances. Several studies have shown that the duration of BBBD depends on the acoustic parameters and the concentrations of MB [21, 31]. One of our own studies revealed that the duration of BBBD can be prolonged by repeated FUS/MB applications [22]. Here, we demonstrated that the time window of FUS-induced BBBD can be shortened by LIPUS alone following FUS/MB application (Fig. 2). The data showed that LIPUS stimulation at 20 min after BBBD induced an obvious decrease in the time window, and there was also a decrease in the above value upon delayed LIPUS stimulation at 1 h after BBBD. Therefore, the data suggest that LIPUS stimulation in the early stage of BBBD will effectively enhance the recovery of the BBB. Further investigations will be required, however, to establish the optimal time window induced by LIPUS for drug delivery in treating various brain diseases.

Cerebral edema has been reported to be one of the major factors for poor outcomes associated with patients with TBI [32]. Effective treatment of vascular brain edema, which is always associated with BBBD, is important to reduce mortality rates [33]. We observed significant differences in brain water content between FUS/MB+LIPUS-treated and FUS/MB-treated rats, providing evidence for the effects of LIPUS on cerebral edema after FUS/MB-induced BBBD (Fig. 3B). FUS/MB-induced BBBD is usually associated with minimal damage to the vasculature or the surrounding brain tissue. This technology cannot be considered to be totally harmless, however, because the fact that erythrocyte extravasation into tissue follows FUS/MB indicates that brain injury has occurred. As such, an effective neuroprotection tool should be developed to ameliorate the possible brain injuries when using this technique for brain diseases in clinical applications. Our results demonstrated that post-injury LIPUS application improved the histological outcomes following FUS/MB-induced BBBD. This improvement was associated with a reduction in hemorrhage, neuronal damage, and apoptotic cell death at 1 day after BBBD (Fig. 4, Fig. 5, and Fig. 6). In this study, exposure of rats to FUS/MB-induced BBBD produced manifestations of moderate to severe TBI, such as hemorrhage, neuron cell death, and apoptosis. Although FUS/MB-induced BBBD is not intended to completely replace the actual TBI model, it could be designed to study TBI. Thus, LIPUS may be a potentially useful method in the treatment of TBI.

The real mechanisms by which FUS and MB exert BBBD are still unknown. Further studies are necessary to address the effects of FUS and MB upon the various transport mechanisms of the BBB. Moreover, investigations aimed at elucidating how LIPUS and the BBB interact at the molecular level are necessary. Results of such studies will increase our understanding of the mechanisms of BBB recovery and also allow a better evaluation of the safety concerns regarding this technique for future clinical applications. This study demonstrated that LIPUS can modulate the time window of FUS-induced BBBD and attenuate the brain injuries following BBBD during drug delivery. As a safe and effective neuroprotection strategy, LIPUS might be proposed as a novel treatment modality for brain injuries after BBBD.

## MATERIALS AND METHODS

### Animals

Male Sprague-Dawley (SD) rats weighing from 280 to 300 g were used in this study. Before ultrasound stimulation, each animal was anesthetized in the prone position by inhalation of 2% isoflurane in 2 l/min oxygen, and the body temperature was maintained at 37°C using a heating pad. The rat heads were mounted on a stereotaxic apparatus (Stoelting, Wood Dale, IL, USA), and the top of the cranium was shaved for ultrasound stimulation. All procedures were approved according to guidelines stipulated by the Animal Care and Use Committee of National Yang Ming University.

### Pulsed ultrasound set-up

Pulsed FUS exposures were generated by a 1.0-MHz, single-element focused transducer (A392S, Panametrics, Waltham, MA, USA) with a diameter of 38 mm and a radius of curvature of 63.5 mm. The half-maximum of the pressure amplitude of the focal zone had a diameter and length of 3 mm and 26 mm, respectively. The experimental ultrasound setup was the same as was used in our previous studies [22, 34]. Ultrasound contrast agent (SonoVue, Bracco International, Amsterdam, the Netherlands) was injected into the tail vein of the rats approximately 15 s before each FUS sonication. This agent contains phospholipid-coated MB with a mean diameter = 2.5 μm, and the concentration = 1−5 × 10^8^ bubbles/ml. The sonication was precisely targeted using a stereotaxic apparatus (Stoelting, Wood Dale, IL, USA). The FUS was delivered to the targeted region in the right hemisphere of each brain at the position of 3.0 mm posterior and 2.5 mm lateral to the bregma, and 5.7 mm below the skull surface. The parameters of the first FUS sonication for BBBD were as follows: an acoustic power of 2.86 W (corresponding to a peak negative pressure of 0.7 MPa) with an injection of 200 μl/kg MB, a pulse repetition frequency of 1 Hz, a duty cycle of 5%, and a sonication time of 60 s. The second sonication with LIPUS alone was applied for the purposes of attenuating BBBD and decreasing tissue damage after the first sonication. LIPUS was applied for a sonication time of 5 min at an acoustic power of 0.51 W (corresponding to a spatial-peak temporal-average intensities (I_SPTA_) of 528 mW/cm^2^).

### Experimental protocol

**Figure 7:**
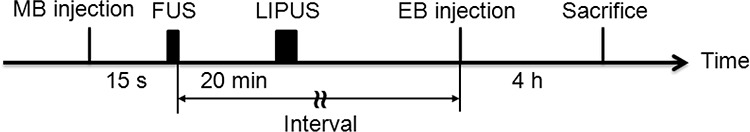
Diagram of experimental procedures for ultrasound stimulations LIPUS exposure was applied at 20 min following the first FUS sonication. EB was injected intravenously at a specific interval after the first FUS sonication.

In the first protocol, twenty-four rats were sonicated with pulsed FUS in the presence of MB (FUS/MB) at an acoustic power of 1.43 or 2.86 W. The rats were injected intravenously with Evans Blue (EB) (Sigma, St. Louis, MO) at various time points (0–4 h) after a single FUS application. Four hours after EB injection, the animals were euthanized and their brains were removed for EB extraction. In the second protocol, all rats were sonicated with FUS at the same acoustic power of 2.86 W. To evaluate the effect of LIPUS treatment on BBBD, rats received a first FUS sonication and were then resonicated with LIPUS alone at an interval of 20 min after the initial treatment. The 20-minute interval between sonications allowed the MB to mostly clear from the circulation before the following LIPUS sonication. Subsequently, the rats were injected intravenously with EB at various time points following LIPUS application (Fig. 7).

For the time window experiment, rats were sonicated twice and then had their EB extravasation evaluated. Three of these rats underwent a second LIPUS sonication at 20 min following the first FUS sonication and were injected intravenously with EB at either 20 min or 1 h after the first sonication. Another three rats were resonicated with LIPUS at 1 h following the first sonication and injected intravenously with EB at either 1 or 2 h after the first sonication. In another experiment, twelve rats were performed to explore the cerebral water content at various time points (1, 4, or 24 h) after FUS-induced BBBD. Another three rats received a second LIPUS sonication at 20 min after the first sonication. Subsequently, the cerebral water content of these rats was evaluated at 4 h after the first sonication. Three rats of each group were used for EB evaluation.

### Assessment of blood-brain barrier permeability

To evaluate the BBB permeability, rats were injected intravenously with EB (Sigma, St. Louis, MO) at a concentration of 100 mg/kg at desired time points after the first FUS application. The animals were sacrificed approximately 4 hours after the EB injection. Rats were perfused with saline via the left ventricle until colorless perfusion fluid appeared from the right atrium. After perfusion and brain removal, the brain was sectioned into three slices from 0 to 6 mm posterior to the bregma, and these slices were mounted on glass slides. The coronal sections were then divided into right and left hemispheres before measuring the amount of EB extravasated. The unsonicated left hemispheres acted as the control. Samples were weighed and then soaked in 50% trichloroacetic acid solution. After homogenization and centrifugation, the extracted dye was diluted with ethanol (1:3), and the amount present was measured using a spectrophotometer (Infinite M200, Tecan, Mechelen, Belgium) at 620 nm. The EB tissue content was quantified via a linear regression standard curve derived from seven concentrations of the dye and was denoted in terms of the amount per gram of tissue.

### Brain water content

Rats were anesthetized and decapitated at three time points (1, 4, and 24 h). Brain water content was measured in the sonicated region of the brain from 0 to 6 mm posterior to the bregma. Brain samples were weighed on an electric analytical balance to obtain the wet weight and then dried at 100°C for 24 h to obtain the dry weight. Brain edema was evaluated by measuring brain water content using the formula of (wet weight-dry weight)/wet weight × 100%. The author who performed the water content evaluation was blinded to the ultrasound parameters but had knowledge of the targeted locations. Four rats of each group were used for water content analysis.

### Histopathology and data analysis

Three rats of each group were prepared for histological observation. The rats were perfused with saline and 10% neutral buffered formalin at one day after first FUS sonication. The brains were removed, embedded in paraffin, and then serially sectioned into 30-μm-thick slices. The slices were stained with hematoxylin-eosin (H&E) to visualize their general cellular structure. Terminal deoxynucleotidyl transferase-mediated dUTP-biotin nick and labeling (TUNEL) staining (DeadEnd Colorimetric TUNEL system, G7130, Promega, Madison, WI, USA) was performed in order to detect DNA fragmentation and apoptotic bodies within the cells. Fluoro-Jade B (FJB) is a polyanionic fluorescein derivative that binds with high sensitivity and specificity to degenerating neurons. Sections were first incubated in a solution of 1% NaOH in 80% ethanol for 5 min and then rehydrated in graded ethanol (75, 50, and 25%; 5 min each) and distilled water. Sections were then incubated in 0.06% KMnO4 for 10 min, rinsed in distilled water for 2 min, and incubated in a 0.0004% solution of FJB (Chemicon, Temecula, CA) for 30 min.

Photomicrographs of 10 μm-thicknesses of the H&E, FJB, and TUNEL-stained tissue sections were obtained using a Mirax Scan digital microscope slide scanner (Carl Zeiss, Mirax 3D Histech) with a Plan-Apochromatic 20/0.8 objective lens. FJB and TUNEL staining was quantified on stained sections from the injury core at the sonicated site. FJB-positive cells were counted by sampling an area of 920 × 860 μm^2^ in three randomly selected, non-overlapping fields with a magnification of 200. Moreover, the same regions were used on each slide across groups. Additionally, the areas showing apoptosis were measured using the Image-Pro Plus software (Media Cybemetics, Silver Spring, MD) in a blinded manner. A total of three tissue sections from each brain were analyzed. The total number of FJB- and TUNEL-positive cells was expressed as the mean number per field of view and in the sonicated resion, respectively.

### Statistical analysis

All values are shown as means ± SEM. Statistical analysis was performed using an unpaired Student *t* test. The level of statistical significance was set at *P* ≤ 0.05.
